# Knowledge, sex, and region associated with primary care providers prescribing adolescents HIV pre-exposure prophylaxis

**DOI:** 10.1038/s41598-023-44165-9

**Published:** 2023-10-08

**Authors:** Garrett Price, Randolph D. Hubach, Joseph M. Currin, Christopher Owens

**Affiliations:** 1 Department of Public Health, College of Health and Human Sciences, Purdue University, West Lafayette, IN USA; 2 Department of Behavioral Sciences and Leadership, United States Air Force Academy, Colorado Springs, CO USA; 3 Department of Health Behavior, School of Public Health, Texas A&M University, 212 Adriance Lab Rd., College Station, TX 77843 USA

**Keywords:** Public health, Drug regulation, Health occupations

## Abstract

Although HIV pre-exposure prophylaxis (PrEP) effectively and safely prevents HIV among adolescents, uptake of PrEP is low. Adolescents must have primary care providers (PCPs) prescribe them PrEP, making PCPs critical actors in PrEP delivery. However, research has primarily investigated determinants of PCPs’ intention to prescribe adolescents PrEP rather than the determinants of performing the behavior itself. We examined the demographic, clinical practice, and implementation determinants of PCPs previously prescribing PrEP to adolescents. PCPs were recruited from a national Qualtrics panel of licensed medical providers in the United States from July 15-August 19, 2022. The Theoretical Domains Framework informed the implementation determinants measured. A multivariable logistic regression was used. PCPs who were more knowledgeable of the CDC guidelines (aOR 2.97, 95% CI 2.16–4.10), who were assigned male at birth (aOR 1.64, 95% CI 1.03–2.59), and who practiced in the Western region (aOR 1.85, 95% CI 1.04–3.30) had greater odds of prior prescribing adolescents PrEP. Provider-based educational interventions should be designed, implemented, and tested to encourage PCPs to prescribe PrEP to eligible adolescents.

## Introduction

In 2019, adolescents and young adults (13–24 years old) accounted for one-fifth of new HIV infections in the United States (US)^1^. The Ending the HIV Epidemic (EHE)^2^ initiative, the Centers for Disease Control and Prevention (CDC)^3^, and the US Preventive Services Task Force (USPSTF)^4^ recommend primary care providers (PCPs) prescribe HIV pre-exposure prophylaxis (PrEP) to patients at risk for contracting HIV and who meet PrEP eligibility requirements, including adolescents. Although adolescents and young adults are at risk of contracting HIV, adolescents and young adults have the greatest unmet need for PrEP among all other age groups^5–9^. Adolescents accounted for 1.5% of all PrEP users in the US in 2012–2017^10^. Given that adolescents need a prescription from a medical provider for PrEP, PCPs are pivotal in increasing PrEP uptake among adolescents who meet eligibility requirements.

There is a growing literature on the prevalence and determinants of PCPs prescribing adolescents PrEP^11–19^. Knowledge about prescribing guidelines, beliefs about safety and patient adherence, parent and confidentiality concerns, self-efficacy, perceived norms, and clinical resources are salient determinants in PCPs’ willingness to prescribe PrEP to eligible adolescents^12,16,19^. However, much of this research has been done before the Food and Drug Administration (FDA) approved PrEP for minors^11–17^, thus investigating hypothetical determinants. Only one survey study^19^ examined the determinants of a national sample of PCPs prescribing PrEP to adolescents post-FDA approval, but the authors examined intention to prescribe rather than actually prescribing PrEP to adolescents. Only one study^16^ investigated determinants of medical providers previously prescribing PrEP to adolescents; however, the authors sampled HIV providers affiliated with the Adolescent Medicine Trials Network for HIV/AIDS Interventions (ATN), a provider group who might be more knowledgeable and comfortable prescribing PrEP to adolescents.

Research is needed to investigate the determinants of PCPs prescribing PrEP to adolescent patients, especially now that the FDA approved PrEP for minors. Such information can be used to identify determinants of actual prescription (rather than intention or willingness), and this information can be used to develop and test provider-based interventions. The purpose of this study was to examine the demographic, clinical practice, and implementation determinants of prior prescription of PrEP to adolescents among a national sample of PCPs. The Theoretical Domains Framework^20^ informed our study. The Theoretical Domains Framework is an integrated framework that combines common health psychology and health service theoretical frameworks. As such, it consists of multiple determinants across ecological levels, such as knowledge at the intrapersonal level, social influence at the interpersonal level, and environmental resources at the organizational level. We chose the Theoretical Domains Framework for four reasons. First, this theoretical framework is commonly used to examine determinants associated with PCPs delivering evidence-based practices^21^. Second, the Theoretical Domains Framework consists of more determinants than previously used theoretical frameworks in PCP PrEP research, such as the three determinants in the Theory of Planned Behavior or the five innovation characteristic determinants in the Diffusion of Innovation^14–16^. Finally, the Theoretical Domains Framework determinants can be measured with a validated questionnaire—the Determinants of Implementation Behavior Questionnaire^22^. Fourth, the Theoretical Domains Framework has been successfully used to investigate the determinants of PCPs’ intention to prescribe adolescents PrEP^19^.

## Methods

### Data collection

This study collected data from July 15th to August 19th, 2022, using a Qualtrics panel of licensed providers in the US^23^. Providers were eligible if they specialized in family medicine or pediatrics. Before joining the Qualtrics provider panel, panel members entered their medical license numbers, and Qualtrics checked their license numbers for accuracy. Qualtrics sent an invitation email to a random sample of panel members to participate in the online cross-sectional survey. The email provided the estimated completion time and incentives. Qualtrics administers its incentive program, where panel members can redeem survey completions with gift cards, cash, and other incentives. Qualtrics automatically removed responses in which the individual did not pass data quality measures, such as attention checks, CAPTCHA, and being outside the average completion time. The Texas A&M University Institutional Review Board (IRB) approved all of the study’s protocols (IRB2022-0695M). This study was conducted in accordance with the Declaration of Helsinki and the university’s IRB. Electronic informed consent was obtained from all participants before they started the questionnaire (*I agree to participate, I disagree to participate*). All participants were 18 years and older. The final sample was 528.

### Measures

#### Demographic characteristics

Participants were asked about their age, assigned sex at birth, gender identity (*male, female, genderqueer, non-binary, transgender, agender, another option*), sexual orientation (*gay or lesbian, bisexual, queer, unsure or questioning, straight or heterosexual, pansexual, asexual, another option*), ethnicity (*yes* or *no* as Hispanic), race (*American Indian or Alaskan Native, Asian or Asian American, Black or African American, Hispanic or Latin American, Middle Eastern or Arab American, Native Hawaiian or Pacific Islander, White, Another option*), and political ideology (1 = *very conservative,* 4 = *middle of the road, and* 7 = *very liberal*). We recoded demographic variables as 0 = female and 1 = male; 0 = gender minority and 1 = heterosexual; 0 = sexual minority and 1 = heterosexual; 0 = person of color and 1 = Non-Hispanic White; political ideology was kept as a continuous variable. We examined these demographic variables because prior research has shown demographic differences in PrEP prescription willingness or prior PrEP prescription, such as providers’ sex and race/ethnicity^24^.

#### Clinical practice characteristics

Participants were asked about their provider type (*physician MD or DO, nurse practitioner, physician assistant*), their zip code and state where their practice or clinic was located, if they had ever taken a sexual history of an adolescent aged 13–18 years old in their current clinic or practice setting (0 = *no* and 1 = *yes*), ordered or recommended an HIV test for an adolescent in their current setting (0 = *no* and 1 = *yes*), ordered or recommended an STI or STD test to an adolescent in their current setting (0 = *no* and 1 = *yes*), and if they had ever prescribed an adolescent aged 13–18 PrEP (0 = *no* and 1 = *yes*). We recoded states in the four US Census Regions^25^. We recoded zip code into rural/urban status using the Index of Relative Rurality^26^. The Index of Relative Rurality is a continuous rural–urban classification scale, where 0.00 is the most urban and 1.00 is the most rural.

#### Theoretical domains framework determinants

Participants were asked how much they agree or disagree with the Theoretical Domains Framework determinants (1 = *strongly disagree*, 2 = *disagree*, 3 = *neither agree nor disagree*, 4 = *agree*, 5 = *strongly agree*). All the determinants consisted of a single item to reduce participant burden, participant time, and survey costs. Items were adapted from the Determinants of Implementation Behavior Questionnaire^22^. The 11 items were knowledge (*I am aware of how to prescribe sexually active adolescents HIV PrEP following the CDC guidelines*), skills (*I have the skills to prescribe sexually active adolescents HIV PrEP*), professional role (*Prescribing sexually active adolescents HIV PrEP is consistent with my professional role*), belief capacity (*I am confident that I can prescribe sexually active adolescents HIV PrEP*), optimism (*I am optimistic when prescribing sexually active adolescents HIV PrEP*), belief consequence (*If I prescribe sexually active adolescents HIV PrEP, it will benefit public health*), attention (*When I need to concentrate to prescribe sexually active adolescents HIV PrEP, I have no trouble focusing my attention*), environmental resource (*In my clinic, all necessary resources are available to prescribe sexually active adolescents HIV PrEP*), social influence (*Most professional in my organization think that I should prescribe sexually active adolescents HIV PrEP*), emotion (*I would enjoy prescribing sexually active adolescents HIV PrEP*), and intention (*I intend to prescribe sexually active adolescents HIV PrEP*).

### Analysis

Data were analyzed using Stata 18. First, descriptive statistics were ran on all variables: number (N), percentage (%), mean (M), and standard deviation (SD). Second, a bivariate analysis was done to find correlations between all variables in relation to prior HIV PrEP prescription (0 = no, 1 = yes). Point-biserial correlations (*r*_*pb*_) were used for continuous variables and chi-square tests (*χ*^2^) for categorical variables. Finally, a multivariable logistic regression was ran using the statistically significant variables from the bivariate analysis, with the outcome variable being the prior prescription of PrEP to an adolescent. We report the adjusted odds ratio (aOR) and 95% confidence intervals (95% CI). For all analyses, a *p*-value (*p*) ≤ 0.05 was statistically significant.

## Results

### Demographic characteristics

Of the 528 respondents, 55.68% were assigned male at birth and 44.32% were assigned female at birth. Most PCPs identified as cisgender (97.92%) and heterosexual (92.61%). About three-quarters were Non-Hispanic White (70.45%), while one-quarter were a person of color (29.55%). The average age of PCPs was 52.47 years old (SD = 10.84). PCPs were nearly equally distributed among political ideologies (M = 4.07, SD = 1.68): 37.13% lean overall conservative, 22.92% lean middle of the road, and 39.96% lean overall liberal. See Table 1 for more information about the frequency of demographic characteristics.

**Table 1:** Demographic and clinical practice characteristics (N = 528).

	N	%	M	SD
Demographic characteristics				
Sex				
Female	234	44.32		
Male	294	55.68		
Gender identity				
Gender minority	11	2.08		
Cisgender	517	97.92		
Sexual orientation				
Sexual minority	39	7.39		
Heterosexual	489	92.61		
Race				
Person of color	156	29.55		
Non-Hispanic White	372	70.45		
Age in years			52.47	10.84
Political ideology			4.07	1.68
Very conservative	24	4.55		
Conservative	103	19.51		
Slightly conservative	69	13.07		
Middle of the road	121	22.92		
Slightly liberal	69	13.07		
Liberal	112	21.21		
Very liberal	30	5.68		
Clinical practice characteristics				
Provider type				
Physician (MD or DO)	510	96.59		
Nurse practitioner	11	2.06		
Physician assistant	7	1.33		
Speciality				
Family medicine	258	48.86		
Pediatrics	270	51.14		
Region				
Northeast	106	20.08		
Midwest	140	26.52		
South	192	36.36		
West	90	17.05		
Rurality			0.29	0.12
Within your current clinic or practice setting, have you ever taken a sexual history with an adolescent aged 13–18 years old?				
No	21	3.98		
Yes	507	96.02		
Within your current clinic or practice setting, have you ever ordered or recommended an HIV test to an adolescent aged 13–18 years old?				
No	40	7.58		
Yes	488	92.42		
Within your current clinic or practice setting, have you ever ordered or recommended an STI or STD test to an adolescent aged 13–18 years old?				
No	23	4.36		
Yes	505	95.64		
Have you ever heard of HIV PrEP before today?				
No	58	10.98		
Yes	470	89.02		
Have you ever prescribed an adolescent aged 13–18 years old HIV PrEP?				
No	370	70.08		
Yes	158	29.92		

### Clinical practice characteristics

Table 1 also depicts the clinical practice characteristics of the sample. Most providers self-reported as physicians (96.59%), with 11 nurse practitioners and seven physician assistants. Providers were equally split among pediatrics (51.14%) and family medicine specialists (48.86%). Providers practiced throughout the US, with 36.36% in the South, 26.52% in the Midwest, 20.08% in the Northeast, and 17.05% in the West. The mean Index of Relative Rurality score was 0.29 (SD = 0.12), indicating most of the sample practiced in an urban county. Nearly all providers took a sexual history of an adolescent patient aged 13–18 within their current clinic or practice setting (96.02%). Similarly, most providers ordered or recommended an HIV test (92.42%) and an STI or STD test (95.64%) to adolescents aged 13–18 within their current clinic or practice setting. Most providers heard of PrEP before participating in the study (89.02%), and 29.92% had prescribed PrEP to an adolescent aged 13–18.

### Theoretical domains framework characteristics

Table 2 shows the frequencies of the 11 Theoretical Domains Framework determinants. All of the Theoretical Domains Framework determinants, but one, had a neutral mean. Belief consequence—also known as instrumental attitude or outcome expectation—had a mean of 4.23 (SD = 0.78), with 88.07% of participants overall agreeing that prescribing PrEP to adolescents will benefit public health. Approximately three-quarters of participants strongly agreed or agreed that prescribing PrEP to adolescents is consistent with their professional role (77.46%, M = 3.93, SD = 0.98), they have the skills to prescribe PrEP to adolescents (73.48%, M = 3.74, SD = 1.08), they are confident or have self-efficacy they can prescribe PrEP to adolescents (70.46%, M = 3.73, SD = 1.03), and they intend to prescribe adolescents PrEP in the future (70.45%, M = 3.81, SD = 0.86). Fifty-seven percent of participants overall agreed that they have clinical resources to prescribe adolescents PrEP (57.76%, M = 3.45, SD = 1.12), and they have no trouble focusing their attention when prescribing adolescents PrEP (57.76%, M = 3.95, SD = 0.97). About half of the participants strongly agreed or agreed that they are aware of how to prescribe adolescents PrEP based on the CDC guidelines (54.92%, M = 3.28, SD = 1.19), and they are optimistic when prescribing adolescents PrEP (50.00%, M = 3.44, SD = 0.91). Nearly one-third of participants overall agreed that they would enjoy prescribing PrEP to adolescents (39.77%, M = 3.28, SD = 0.94), and most professionals in their organization think that they should prescribe adolescents PrEP (37.12%, M = 3.27, SD = 0.96).

**Table 2:** Theoretical domains framework characteristics (N = 528).

Theoretical domains framework determinant	N	%	M	SD
Knowledge: I am aware of how to prescribe sexually active adolescents HIV PrEP following the CDC guidelines			3.28	1.19
Strongly disagree	50	9.47		
Disagree	105	19.89		
Neither agree nor disagree	83	15.72		
Agree	225	42.61		
Strongly agree	65	12.31		
Skill: I have the skills to prescribe sexually active adolescents HIV PrEP			3.74	1.08
Strongly disagree	32	6.06		
Disagree	46	8.71		
Neither agree nor disagree	62	11.74		
Agree	276	52.27		
Strongly agree	112	21.21		
Professional role: prescribing sexually active adolescents HIV PrEP is consistent with my professional role			3.93	0.98
Strongly disagree	22	4.17		
Disagree	22	4.17		
Neither agree nor disagree	75	14.20		
Agree	263	49.81		
Strongly agree	146	27.65		
Belief capacity: I am confident that I can prescribe sexually active adolescents HIV PrEP			3.73	1.03
Strongly disagree	26	4.92		
Disagree	42	7.95		
Neither agree nor disagree	88	16.67		
Agree	266	50.38		
Strongly agree	106	20.08		
Optimism: I am optimistic when prescribing sexually active adolescents HIV PrEP			3.44	0.91
Strongly disagree	16	3.03		
Disagree	51	9.66		
Neither agree nor disagree	197	37.31		
Agree	212	40.15		
Strongly agree	52	9.85		
Belief consequence: if I prescribe sexually active adolescents HIV PrEP, it will benefit public health			4.23	0.78
Strongly disagree	8	1.52		
Disagree	5	0.95		
Neither agree nor disagree	50	9.47		
Agree	260	49.24		
Strongly agree	205	38.83		
Attention: when I need to concentrate to prescribe sexually active adolescents HIV PrEP, I have no trouble focusing my attention			3.95	0.97
Strongly disagree	21	5.68		
Disagree	19	17.42		
Neither agree nor disagree	82	19.13		
Agree	251	42.23		
Strongly agree	155	15.53		
Environmental resource: in my clinic, all necessary resources are available to prescribe sexually active adolescents HIV PrEP			3.45	1.12
Strongly disagree	30	5.68		
Disagree	92	17.42		
Neither agree nor disagree	101	19.13		
Agree	223	42.23		
Strongly agree	82	15.53		
Social influence: most professionals in my organization think that I should prescribe sexually active adolescents HIV PrEP			3.27	0.96
Strongly disagree	20	3.79		
Disagree	74	14.02		
Neither agree nor disagree	238	45.08		
Agree	138	26.14		
Strongly agree	58	10.98		
Emotion: I would enjoy prescribing sexually active adolescents HIV PrEP			3.28	0.94
Strongly disagree	25	4.73		
Disagree	58	10.98		
Neither agree nor disagree	235	44.51		
Agree	162	30.68		
Strongly agree	48	9.09		
Intention: I intend to prescribe sexually active adolescents HIV PrEP in the future			3.81	0.86
Strongly disagree	14	2.65		
Disagree	15	2.84		
Neither agree nor disagree	127	24.05		
Agree	275	52.08		
Strongly agree	97	18.37		

*N* number, *%* percentage, *M* mean, *SD* standard deviation.

### Bivariate analyses

The bivariate analyses are listed in Table 3. All of the 11 Theoretical Domains Framework determinants were positively correlated with prior prescription of PrEP to an adolescent: knowledge (*r*_*pb*_ = 13.24, *p* < 0.001), intention (*r*_*pb*_ = 0.27, *p* < 0.001), skills (*r*_*pb*_ = 0.27, *p* < 0.001), belief capacity or self-efficacy (*r*_*pb*_ = 0.27, *p* < 0.001), emotion (*r*_*pb*_ = 0.15, *p* < 0.01), optimism (*r*_*pb*_ = 0.25, *p* < 0.01), professional role (*r*_*pb*_ = 0.24, *p* < 0.01), environmental resource (*r*_*pb*_ = 0.23, *p* < 0.01), social influence or perceived norm (*r*_*pb*_ = 0.23, *p* < 0.001), belief consequence or outcome expectation (*r*_*pb*_ = 0.15, *p* < 0.001), and attention (*r*_*pb*_ = 0.13, *p* < 0.001).

**Table 3:** Correlation for prior prescription of PrEP to an adolescent (N = 528).

	*r*_*pb*_	*χ*^2^
Age	− 0.03	
Political ideology	− 0.03	
Urbanity/rurality	− 0.06	
Knowledge	0.45***	
Skills	0.27***	
Professional role	0.24***	
Belief capacity	0.27***	
Optimism	0.25***	
Belief consequence	0.15***	
Attention	0.13***	
Environmental resource	0.23***	
Social influence	0.23***	
Emotion	0.15***	
Intention	0.27***	
Sex (ref: female)		13.24***
Gender (ref: gender minority)		0.22
Sexual orientation (ref: sexual minority)		2.47
Race (ref: person of color)		3.77
Provider speciality (ref: family medicine)		10.70**
Northeast		3.35
Midwest		0.70
South		0.09
West		6.47*
Past sexual history taking (ref: no)		2.55
Past HIV testing (ref: no)		10.38***
Past STI or STD testing (ref: no)		5.17*

*r*_*pb*_ Point-Biserial correlation, *χ*^*2*^ chi-square test.

*p < .05, **p < .01 ***p < .001.

The only demographic variable that was correlated with prior prescription of PrEP was sex assigned at birth, with prior prescription being positively associated with assigned male at birth (*χ*^2^ = 13.24, *p* < 0.001). Regarding clinical practice variables, prior prescription of PrEP to adolescents was positively correlated with prior ordering or recommending of an HIV test to an adolescent (*χ*^2^ = 10.38, *p* < 0.001), prior ordering or recommending of an STD/STI test to an adolescent (*χ*^2^ = 5.17, *p* = 0.05), prior hearing about PrEP (*χ*^2^ = 27.82, *p* < 0.001), if the provider was located in the Western region of the US (*χ*^2^ = 6.47, *p* < 0.05), and if the provider specialized in pediatrics (*χ*^2^ = 10.70, *p* < 0.01).

### Logistic regression

Table 4 presents results from the multivariable logistic regression. The multivariable logistic regression model is statistically significant, *χ*^2^ (16) = 156.68, *p* < 0.001. The model explained 36.4% (Nagelkerke R^2^) of the variance in prior PrEP prescriptions. The only Theoretical Domains Framework that was associated with previously prescribing adolescents PrEP was knowledge of the CDC guidelines (aOR = 2.97, 95% CI 2.16–4.10). The model showed that providers assigned male at birth have 1.64 times greater odds of previously prescribing PrEP to adolescents than providers assigned female at birth (aOR = 1.64, 95% CI 1.03–2.59). The model depicted providers located in the Western region of the US had 1.85 times greater odds of having prescribed an adolescent PrEP as opposed to the other regions (aOR = 1.85, 95% CI 1.04–3.30).

**Table 4:** Multivariable logistic regression for prior prescription of PrEP to adolescents (N = 528).

	aOR	Std. E	95% CI
Sex (ref: female)	1.63	0.39	[1.03, 2.59]
Pediatrics (ref: family medicine)	1.14	0.27	[0.72, 1.81]
HIV testing (ref: no)	5.11	5.08	[0.73, 35.90]
STI testing (ref: no)	0.75	0.93	[0.07, 8.50]
West	1.85	0.55	[1.04, 3.30]
Knowledge	2.97	0.49	[2.16, 4.10]
Skills	1.06	0.22	[0.70, 1.60]
Professional role	1.19	0.25	[0.79, 1.80]
Belief capacity	0.82	0.19	[0.52, 1.29]
Optimism	1.33	0.24	[0.94, 1.88]
Belief consequence	1.02	0.21	[0.69, 1.52]
Attention	0.90	0.13	[0.68, 1.20]
Environmental resource	0.93	0.13	[0.70, 1.23]
Social influence	1.00	0.16	[0.73, 1.35]
Emotion	0.83	0.14	[0.60, 1.17]
Intention to prescribe PrEP	1.38	0.29	[0.91, 2.09]
Constant	0.00	0.00	[0.00, 0.01]

*aOR* adjusted odds ratio, *Std. E* standard error; *95% CI* 95% confidence interval.

## Discussion

This is the first national study to analyze the demographic, clinical practice, and implementation determinants of PCPs prior PrEP prescription behaviors to adolescents after the FDA approved PrEP for minors. Approximately 30% of our sample reported they ever prescribed PrEP to an adolescent patient, and this is similar other studies^12,19^ that reported about one-third of providers in their sample ever prescribed PrEP to an adolescent. Our study was informed by the Theoretical Domains Framework^20^, a common implementation framework used to examine the implementation factors of providers delivering an evidence-based practice^21^. One variable per the Theoretical Domains Framework (knowledge), demographic characteristics (sex assigned at birth), and clinical practice characteristics (region) were significant. Our findings have important implications for behavioral-based education, theory, and practice on prescribing PrEP to adolescents.

Knowledge about the CDC’s guidelines has been shown to be a significant determinant in PCPs’ intention to prescribe adolescents PrEP^12,16,19^, and our finding suggests that knowledge extends into actually prescribing adolescents PrEP. This illustrates the need for PCPs to be educated about the CDC’s guidelines, as knowledge is associated with PCPs prescribing PrEP to adult patients^27–33^. Multiple on-site trainings^34^ might be more effective in increasing PrEP prescription behavior than single session trainings^35^. Moreover, medical schools could incorporate the CDC’s guidelines into their medical education and medical residency programs, as medical students report PrEP is not included in their training^36–39^. Future research should examine what facilitators and barriers medical school, residency, and clinic leaders have in providing PrEP training.

While our study found that the only statistically significant Theoretical Domains Framework determinant was knowledge, prior research has highlighted several other determinants, including PCPs’ attitudes, self-efficacy and skills, professional roles, perceived norms, and clinical environment resources^11–19^. This might not be surprising, as providers who have more positive attitudes about an evidence-based practice and more clinical resources are more likely to deliver that evidence-based practice. A possible explanation is that knowledge might be a moderator or mediator for other determinants. Indeed, a study^33^ utilizing the Information-Motivation-Behavioral Skill Model (IMB) demonstrated that PrEP-related information directly affected the PrEP-related attitudes and skills among PCPs. While the Theoretical Domains Framework explained 42% of the variance of PCPs’ intention to prescribe PrEP to their patients^19^, the IMB explained 50% of the variance^33^. Future studies might use the IMB as their theoretical framework in investigating the determinants of PCPs prescribing PrEP to teens.

We found that PCPs who are males at birth prescribed PrEP to adolescents more than PCPs females at birth. Nearly all of our sample were cisgender. While Owens et al.^19^ found sex was not a determinant of PCP’s willingness to prescribe PrEP to adolescents, Leech et al.^40^ found females were less likely than males to have prescribed PrEP to adult male patients. We hypothesize three reasons for why male PCPs prescribed adolescents PrEP more than female PCPs. First, adolescent^41^ and adult patients^42^ prefer to seek PCPs who share in the patient’s sex. Because HIV affects males at birth more than females at birth in the US, male PCPs may have more interactions than female PCPs with PrEP-eligible and PrEP-seeking patients. Second, systematic reviews have noted that male providers adopt new prescriptions earlier than female providers^43,44^. Third, more family medicine/general practice physicians are males (57.7%) than females (42.3%)^45^.

Our study showed that PCPs in the Western region had 1.85 greater odds of prescribing adolescents PrEP in the past. This result corroborates with the literature that consistently shows PrEP use is higher in the Northeastern and Western regions than in the Southern and Midwestern regions^5–7,9^. It is likely that PrEP prescriptions are higher in the Western region due to the availability of PrEP clinics and PrEP providers^46,47^ as well as policies such as Medicaid expansion and PrEP drug assistance programs^5,6,48^. For example, the concerted efforts by public health entities in California expanded access to PrEP through community pharmacies, Medicaid expansion, and PrEP assistance programs^49,50^. Public health and HIV entities could advocate for their state legislatures to adopt these PrEP access policies.

Moreover, Western and Northeastern states tend to allow minors to access HIV prevention services, including PrEP, without parental/guardian permission^51,52^. Thirty-four states and D.C. allow minors to consent to HIV prevention services; however, only 18 of these address confidentiality protections. Public health, HIV, and adolescent health entities might want to educate providers about their state’s minor consent/confidentiality laws, as providers tend to be unfamiliar with their state’s law, given the wide variability of minor consent laws^53,54^. Public health, HIV, and adolescent health entities might want to advocate for their state legislatures to increase the legal capacity of minors to consent to HIV prevention services. Research is necessary to understand the facilitators, barriers, and strategies that effectively change state laws that expand HIV prevention services to adolescents.

### Limitations

This study has limitations. First, our sample was primarily composed of physicians, with nurse practitioners and physician assistants making up 3% of the sample. More research is necessary to examine the implementation determinants of nurse practitioners in states where they are given full prescriptive authority. Second, we utilized cross-sectional data, meaning we can claim correlations but not causations. Third, our patient vignette did not include the adolescent’s demographic characteristics. Similarly, participants provided their agreement and disagreement to questions about PrEP delivery to adolescents aged 13–18 years old rather than a specific age (e.g., 16 years old) or an age range (15–17). However, some states define minors as those who are 18–21^51,52^. Medical providers have noted in prior research that patient characteristics and providers’ perceptions of patients play a part in their intention to prescribe and in their actual prescribing of PrEP to adolescents^12–15,17,18^. Fourth, our study only analyzed prior prescription of PrEP and not other USPSTF recommendations regarding PrEP identification, discussion, and care, such as taking an HIV risk assessment to identify adolescents at risk of contracting HIV, educating adolescent patients and their parents about PrEP, or discussing the risks and benefits of the different PrEP medications and modalities (e.g., Truvada® v. Descovy® v. Apretude®; daily oral v. long-acting injectable)^4^.

## Conclusion

This study, utilizing data collected from a national sample of PCPs, demonstrates that PCPs who are more knowledgeable about the CDC’s guidelines had greater the odds of prescribing adolescents PrEP. It might be beneficial to implement provider-targeted PrEP training and education programs to medical students, medical residents, and medical providers. Additionally, findings from our study suggest that scientists who study provider PrEP-related behavior might want to use theoretical frameworks that posit that information affects other constructs. We might have seen more PCPs prescribing PrEP to adolescents in the Western region because of state policies. Public health, HIV, and adolescent health entities might want to advocate state legislatures to adopt laws that allow adolescents to consent to HIV prevention services. Such education and legal interventions might increase the rates in which PCPs prescribe PrEP to eligible adolescents, therefore decreasing HIV rates among adolescents and young adults.

## Data Availability

The datasets generated during and/or analyzed during the current study are available from the corresponding author on reasonable request.
